# Concepts of lines of therapy in cancer treatment: findings from an expert interview-based study

**DOI:** 10.1186/s13104-024-06789-6

**Published:** 2024-05-15

**Authors:** Lisa Falchetto, Bernd Bender, Ian Erhard, Kim N. Zeiner, Jan A. Stratmann, Florestan J. Koll, Sebastian Wagner, Marcel Reiser, Khayal Gasimli, Angelika Stehle, Martin Voss, Olivier Ballo, Jörg Janne Vehreschild, Daniel Maier

**Affiliations:** 1 Institute for Digital Medicine and Clinical Data Science, Goethe University Frankfurt, Faculty of Medicine, Frankfurt, Germany; 2 German Cancer Consortium (DKTK), partner site Frankfurt/Mainz and German Cancer Research Center (DKFZ), Heidelberg, Germany; 3 Department for Dermatology, Venerology and Allergology, University Hospital Frankfurt, Frankfurt, Germany; 4 Department of Urology, University Hospital Frankfurt, Frankfurt, Germany; 5 PIOH Praxis Internistischer Onkologie und Hämatologie, Cologne, Germany; 6 Clinic for Gynecology and Obstetrics, University Hospital Frankfurt, Frankfurt, Germany; 7 Department for Internal Medicine 1, University Hospital Frankfurt, Frankfurt, Germany; 8 Department Neuro-Oncology, University Hospital Frankfurt, Frankfurt, Germany; 9 Department I of Internal Medicine, University Hospital of Cologne, Cologne, Germany; 10 German Center for Infection Research (DZIF) partner site Bonn Cologne, Cologne, Germany; 11 Medical Department 2 (Hematology/Oncology), Center for Internal Medicine, University Hospital Frankfurt, Goethe University Frankfurt, Frankfurt, Germany

**Keywords:** Lines of therapy, cancer treatment, Therapy planning, Expert interview

## Abstract

**Objective:**

The concept of lines of therapy (LOT) in cancer treatment is often considered for decision making in tumor boards and clinical management, but lacks a common definition across medical specialties. The complexity and heterogeneity of malignancies and treatment modalities contribute to an inconsistent understanding of LOT among physicians. This study assesses the heterogeneity of understandings of the LOT concept, its major dimensions, and criteria from the perspective of physicians of different specialties with an oncological focus in Germany. Semi-structured expert interviews with nine physicians were conducted and evaluated using qualitative content analysis.

**Results:**

Most interviewees agreed that there is no single definition for LOT and found it difficult to explicate their understanding. A majority of experts stated that they had already encountered misunderstandings with colleagues regarding LOT and that they had problems with deciphering LOT from the medical records of their patients. Disagreement emerged about the roles of the following within the LOT concept: maintenance therapy, treatment intention, different therapy modalities, changing pharmaceutical agents, and therapy breaks. Respondents predominantly considered the same criteria as decisive for the definition of LOT as for a change in LOT (e.g., the occurrence of a progression event or tumor recurrence).

**Supplementary Information:**

The online version contains supplementary material available at 10.1186/s13104-024-06789-6.

## Introduction

While clinical oncology considers line of therapy (LOT) essential information for therapy planning, the field lacks a homogeneous understanding of the concept, as well as clear and consistent criteria for its classification [1]. Especially in real-world data-based research, it is often unclear whether a certain therapy is still part of an LOT; and often, conflicting interpretations lead to misunderstandings in information exchange about therapy progression [1]. Existing approaches, for standardizing the classification of LOT either focus on patterns proposed by guidelines (e.g., drug administration period, first-line termination) or on drug administration sequences [2–6]. However, other issues related to the LOT concept remain largely unclear. For example, the roles of maintenance therapies and local therapy modalities have not yet been discussed [1].

This expert-interview study aims to provide a better conceptual understanding of the defining criteria of LOT for solid and non-solid cancers. Therefore, it may contribute to identifying unclear aspects of the LOT concept and avoiding misunderstandings in communication about LOTs, especially between physicians of different medical disciplines. Concerning the rapidly developing field of real-world cancer research, data augmentation strategies and feature engineering require *empirically validated concepts* to obtain reliable evidence from observational data. More specifically, investigating the conceptual understanding of LOTs will help us build a rule-based framework for LOT classification within the Clinical Communication Platform of the German Cancer Consortium (DKTK).

## Methods

### Sample

The study’s target group was physicians from various specialties with an oncological focus, working in either university hospitals or private practice. Physicians from the University Hospital Frankfurt and private practices were contacted by e-mail. In total, nine were interviewed. Their varied specialties included neuro-oncology, pulmonology, hematology and medical oncology, urology, dermatology, and gynecological oncology, as well as one resident specialist in internal medicine with a focus on hematology and oncology. The interviewees’ professional experience ranged from 3.5 to 29 years and most had experience in treating both solid and non-solid malignancies.

### Instrument

Qualitative expert interviews [7, 8] were conducted by posing open questions within a semi-structured framework [9]. An interview manual delineated this framework and was developed based on existing literature about oncological LOTs and associated concepts (see Additional File 1). Before the interviews, the interview manual was pre-tested with an experienced oncologist and adjusted accordingly. Each participant declared their consent before the interview. Confidentiality and anonymity of participants’ responses and information were assured. The first part of the interview manual asked about the interviewee’s underlying understanding of LOTs and the relevant criteria for their definition. Subsequently, questions concerning misunderstandings in interactions with colleagues were posed to determine whether there are frequent uncertainties in the use of the LOT concept and, if so, what reasons may underlie this situation. Next, the interviewer asked about how specific criteria, picked out of the literature, related to the definition of LOT. These included the influence of treatment intention, the role of maintenance therapy, and local therapies. Another focus of the interviews was how the interviewees judged the relationship of both changes in drug regimen and therapy breaks to the definition of LOT.

### Data collection/conduct of interviews

The expert interviews were conducted between June 1 and July 17, 2022 via video conference and in German. They lasted between 10 and 25 minutes with an average duration of approximately 18 minutes. The interviews were recorded and transcribed using the ExpressScribe Pro software (Version 10.17).

### Data analysis

The interviews were analyzed using methods of qualitative content analysis as described in Mayring [10] and the software MaxQDA Analytics Pro 2022 (release 22.2.0). A system for coding the interview material was developed based on literature research conducted before the interviews.

## Results

Since the interviews were conducted in German, we provide an English translation of selected quotes. Table 1 contains the main topics and sub-topics of the interview, as well as exemplary quotes from the interviewees.

**Table 1:** Topics, sub-topics and quotes from the interviews

Topics and sub-topics	Descriptors
**Topic 1. Understanding of the LOT**	
Understanding of the LOT: lack of uniform definition	“After all, we had discussed this many times. A delineated therapy that is carried out until the disease progresses or until it is discontinued – either because of side effects or because it was terminated in a regular manner.”
Understanding of the LOT: problem of articulating one’s understanding of LOT	“[…] – that is a bit difficult to describe.”
**Topic 2. Problems in everyday work**	
Problems in every work: misunderstandings with colleagues	“[…] when it comes to categorizing it somehow so that it is standardized and applicable across multiple centers, yes there existed discrepancies in the particular considerations.”
Problems in every work: uncertainties in determining LOT	“[…] I really have to get familiarize myself with some patients and think through the questions: is this still maintenance therapy or had it already been recurrence therapy, when I only see the diagnosis notebook.”
**Topic 3. Treatment intention**	
Influence of treatment intention on definition and determination of LOT	“With a curative therapy option […] you shouldn’t have any progression under therapy, after all. So that´s why the definition [of line of therapy] does differ somewhat – palliative versus curative.”
**Topic 4. Maintenance therapy**	
Maintenance therapy: as continuation of the previous LOT	“I would probably count maintenance therapy as part of that – if it’s sort of logically linked to the therapy that was administered before it.”
Maintenance therapy: uncertainty with regard to assignment to a previous LOT	“Yes, that’s difficult, too. […] But if it’s a completely different type of substance now, then it becomes even more difficult again.”
**Topic 5. Local vs. systemic therapy modalities**	
Local vs. systemic therapy modalities: inclusion of both in LOT	“[…] curative therapy concepts are often multimodal. That means you operate, you give another chemo[therapy], you may even give another immunotherapy. This means that the therapy concept contains various components. Personally, I would perceive it all as one line. […]”
Local vs. systemic therapy modalities: systemic therapy modalities as sole determiners of the LOT concept	“In my opinion the therapy line is primarily defined by the systemic therapies. The local therapies are rather something supplementary that carried out additionally or – as the case may be – primarily in addition for symptom relief. Local therapies can also be used to achieve a response, but are not usually mentioned as a line of therapy.”
**Topic 6. Change of LOT**	
Change of LOT: progression and recurrence as indicators of change in LOT	“[…] it’s tied to recurrence and progression. Any chemotherapy we initiate will then be the next line of therapy.”
Change of LOT: adverse effects and toxicity as indicators in change of LOT	“Dropping an active substance, I would always see as being due to toxicity […] I would never call that a new line of therapy […]”
Change of LOT: planned end of a therapy is indicator of change in LOT	“A therapy is over for me when the designated timeframe ends – I would look at it temporally.”
Change of LOT: patient’s wishes as indicator of change in LOT	“Or with patients’ wishes, you have that from time to time, that someone does not want to continue for various reasons. And then, of course, the therapy is concluded for the time being.”
Change of LOT: disagreement on the role of changes in pharmaceutical agent	“[…] whereas the addition of a new agent – strictly speaking, it would have to be considered a new line of therapy, although it is also difficult in terms of definition.”
**Topic 7. Therapy breaks**	
Therapy break: importance of break duration	“Normally, a short therapy break is due to tolerance problems or something similar – then one would say it’s rather the same line of therapy. [In certain cases of colon cancer, there it is] somewhat more difficult when much longer therapy breaks of several months, or sometimes perhaps even up to a year, are made. And then, as a rule, one would not continue with the same therapy.”
Therapy break: importance of tumor aggressiveness	“It depends on the time and the entity and the aggressiveness of the tumor.”
Therapy break: importance of the therapy started after the break	“As long as the same therapy started again after the break, it is definitely the same line of therapy in my view. If the therapy is switched after a long break, it’s a second line of therapy to me.”
Therapy break: importance of disease progression or recurrence	“[…] in principle, if no recurrence has occurred and it is perhaps even the same substance that you’re picking up again, then I would consider it one line of therapy, regardless of how long the break was. “

### LOT definition and misunderstandings

Most interviewees confirmed that there was no common understanding of LOT and that they had difficulties explicating their own understanding of the concept. Furthermore, four of the interviewees reported misunderstandings with colleagues regarding LOTs and seven reported that they experienced uncertainties in their clinical practice when defining an LOT. For instance, if care for a patient was delivered by multiple centers, misunderstandings concerning LOT progression frequently occurred, because involved persons lacked a common understanding:

“[…] when it comes to categorizing it somehow so that it is standardized and applicable across multiple centers, yes there existed discrepancies in the particular considerations.” (Expert interview (E)05).

### Treatment intention

Six interviewees said that treatment intention (curative vs. palliative) is important in the choice of therapy. Consequently, treatment intention is also relevant to LOT planning. Three experts expressed that LOT is especially relevant and established in the palliative setting:

“With a curative therapy option, […] you shouldn’t have any progression under therapy, after all. So that’s why the definition [of the line of therapy] does differ somewhat – palliative versus curative.” (E03).

### Maintenance therapy

Starting a maintenance therapy to control a tumor after chemotherapy was predominantly not considered an indicator for a change in LOT, since usually only part of the medication regimen is discontinued for maintenance, while the rest remains the same. However, interviewees also said that maintenance therapy can include an entirely new pharmaceutical agent, which would, in turn, complicate the delineation between LOT:

“Yes, that’s difficult, too. I would probably count maintenance therapy as part of that – if it’s sort of quasi-logically linked to the therapy that was administered before it. But if it’s a completely different type of substance now, then it becomes more difficult again.” (E03).

### Local therapies vs. systemic therapies

Six of the physicians interviewed opined that a LOT can contain both local and systemic therapies. However, some participants stated that beginning a new local therapy would not lead to a change of LOT, in contrast to beginning a new systemic therapy. Meanwhile, in contrast to the other six, three physicians emphasized that only systemic therapies can constitute a LOT:

“In my opinion, the therapy line is primarily defined by the systemic therapies. The local therapies are rather something supplementary that is carried out additionally, or – as the case may be – primarily in addition to symptom relief. Local therapies can also be used to achieve a response, but are not usually mentioned as a line of therapy.” (E06).

### Change of LOT

All interviewees said that the LOT must be changed if tumor progression or disease relapse occurs or if therapy response fails. Six interviewees considered the occurrence of adverse effects (e.g., severe toxicity) a significant criterion for the decision to change an LOT. Only three interviewees saw the addition of a new pharmaceutical agent as resulting in a change of LOT:

“Dropping an active substance, I would always see as being due to toxicity or at the patient’s request – so actually owed to toxicity. That is, I would never call that a new line of therapy, whereas the addition of a new agent – strictly speaking, it would have to be considered a new line of therapy, although it is also difficult in terms of definition.” (E09).

The other seven interviewees only considered the introduction of new pharmaceutical agents a change in LOT if the treatment intention changed as well, or if a recurrence or progression occurred. Only the replacement of one drug with another of the same class (e.g., cisplatin with carboplatin) was not considered a change of LOT by anyone.

### Therapy breaks

There were also ambiguous opinions regarding the role of breaks in therapy for the classification of LOT. On the one hand, the length of the break was considered decisive, whereas on the other hand, it was said that the therapy following the break was more important. Additionally, some viewed breaks in therapy as important for the classification of LOT in the event of a relapse or progression:

“[…] In principle, if no recurrence has occurred and it is perhaps even the same substance […] then I would consider it one line of therapy, regardless of how long the break was.” (E01).

If the break was unplanned, it was considered a significantly more important criterion for a change in LOT than if it was part of the therapy concept.

## Discussion

The expert interviews in this study largely confirmed that there is no common understanding of the LOT concept or its defining criteria. The interview material suggests that individual backgrounds in differing medical disciplines may influence views on and understandings of LOT. This potential context dependency of the LOT concept also appears consistent with heterogeneous working definitions of LOT in different real-world studies of distinct cancer entities [1, 11, 12].

However, it appeared that a LOT was considered a therapeutic concept with start- and endpoints that is focused on systemic therapies, although it may also contain additional treatment modalities. If included in the LOT, such non-systemic modalities would be selected based on individual patient and disease characteristics, and terminated if certain events (e.g., tumor progression) occurred.

There was evident uncertainty about the role of adjuvant and maintenance therapy and whether they should be regarded as an LOT together with the preceding (systemic) therapy. Also, no prevailing opinion could be identified on the questions of whether treatment intention (curative vs. palliative) and therapy breaks were integral to defining LOTs. Furthermore, experts held differing opinions on which changes in the administered drug regimen would initiate a change in LOT.

In the literature, however, individual approaches for standardizing the criteria for a change in LOT exist in the following cases: the termination of a LOT is indicated in the event of treatment discontinuation, addition of a new, non-equivalent agent, interruption of treatment, clinical progression of the disease, or death of the patient [2, 3]. The interviewees were also nearly unanimous on these criteria: all considered tumor progression and recurrence decisive for a change in LOT; six experts highlighted the occurrence of side effects or relevant toxicity; three mentioned the scheduled end of therapy; and one cited patients’ wishes. Only some of the interviewees considered a change in pharmaceutical regimen a factor in identifying a change in LOT, while replacement of one drug with another from the same class was not viewed as altering the LOT.

The interviews both identified tumor recurrence and progression as LOT-relevant events and raised questions about the nature of their role. Recurrence and progression during therapy breaks, as well as the length of the break and the treatment thereafter, were considered relevant factors for a change in LOT. In two interviews, although the participants initially identified recurrence and progression as indicators for a change in LOT, their further comments appeared to contradict this standpoint. This apparent inconsistency should be investigated in future research.

Seven interviewees considered treatment intention relevant to LOT. Predominantly, interviewees considered the adoption of maintenance therapy as a continuation of an ongoing LOT. However, it remains unclear whether changes in the dosage or interval of drug administration during maintenance therapy imply a change in LOT. Six interviewees said that both local and systemic therapy modalities should be included in characterizations of LOT, although previous research excluded local modalities [1, 13–15].

While similar approaches to standardizing the duration of a LOT [2] and first-line therapy [2, 3] exist, it is not clear whether the definition of LOT can be standardized across disciplines as well as tumor entities. Nevertheless, a cross-disciplinary standard definition of the LOT concept should be targeted.

### Limitations

This study exhibits the following limitations:

- Qualitative expert interviews were only feasible for a small sample (*n* = 9) of oncological experts, most of whom were located at a single center (eight out of nine). While the study delivers highly granular insights, this approach precludes generalization of the findings. Therefore, subsequent research must evaluate the qualitative insights leaned from this study in larger and more representative samples.
- The interviewees had varying degrees of professional experience and different specialties, making direct comparisons of experience and assessments regarding oncological LOT difficult. However, this was intentional to obtain the widest possible range of assessments regarding the broad topic under investigation.
- No triangulation in the form of using multiple and diverse data sources, perspectives, locations, or theories took place in conducting the study. Such methods can help to mitigate subjective bias resulting from the explicit focus on one’s own data [16].

### Electronic supplementary material

Below is the link to the electronic supplementary material.

**Additional file 1.** Interview manual with all instructions and questions.

## Data Availability

Details on the data and materials related to the study may be available upon reasonable request from Bernd Bender (B.Bender@med.uni-frankfurt.de).

## Abbreviations

DKTK: German Cancer Consortium
E: Expert interview
LOT: Line of therapy
