# Supplementary material for: Concepts of lines of therapy in cancer treatment: findings from an expert interview-based study
Source: BMC Res Notes. 2024 May 15;17:137. doi: 10.1186/s13104-024-06789-6 (PMC11094945; doi:10.1186/s13104-024-06789-6)
Supplement: Supplementary file 1 — Additional file 1. Interview manual with all instructions and questions. [file 13104_2024_6789_MOESM1_ESM.docx]

**Interview manual for concepts of lines of therapy**

**Information**

Interview with:

Date:

Location:

Duration:

Declaration of consent:

**Preliminary remarks on the interview**

Thank you [Mr./Ms. XY] for taking the time to participate in this interview. I would first like to briefly explain why I would like to conduct this interview with you. The aim of my doctoral thesis is to find out whether and to what extent there are different understandings of the term and definition of lines of therapy among physicians who work in the care of oncological patients. It is important that there are no right or wrong answers on your part in this interview, as I want to explore the individual understandings of therapy lines.

The interview will take about 20, maximum 30 minutes. I have created an interview manual so that I do not leave out any important questions. However, it is perfectly all right if we deviate from it from time to time, as some topics will be more important to you than others.

All information you provide to me will be treated confidentially and the results will be anonymized. All data will only be used for the purpose of the research project.

I would like to record the interview so that I can devote my full attention to you during the interview and no information is lost. Do you agree to an audio recording? [Start recording]

**Start of interview**

**Understanding the term**

1. In the treatment of oncological patients, we often talk about lines of therapy. What do you understand by the term "line of therapy"?
   1. You said that XYZ is a central criterion that defines the start of a new line of therapy. Can you explain this in more detail?
   2. In your opinion, what are other important criteria that should be used to define lines of therapy?

**Problem**

1. Have you ever experienced a misunderstanding about the concept of lines of therapy when discussing a case with colleagues? If so, what was the reason?

**Treatment intention and maintenance therapy**

1. Depending on the stage of the tumor and the patient's state of health, there are curative or palliative treatment options. In your opinion, are there differences in the definition of the line of therapy, depending on the treatment intention? If so, which ones?
2. Maintenance therapy also plays an important role in the treatment of malignant tumors. How would you define maintenance therapy and what influence does it have on the classification of lines of therapy?

**Local vs. systemic therapy modalities**

1. Generally speaking, there are local and systemic treatment options in cancer therapy. In your opinion, what role do local therapeutic modalities, such as surgery/TACE/radiotherapy, and what role do systemic therapies, such as chemotherapy or hormone therapy, play in defining a line of therapy?

**Change of line of therapy**

1. Opinions regarding the end of a line of therapy diverge widely. How do you judge when a line of therapy has ended?
2. In the following, we will focus on systemic drug therapies. What influence does a change in the substance regimen have on the line of therapy?
   1. What effect does the replacement of a drug with a drug from the same class (e.g. cisplatin with carboplatin) have on the line of therapy?
   2. How would you assess the situation if a targeted substance (e.g. a monoclonal antibody) is added to an existing systemic drug therapy?

**Therapy break**

1. In the treatment of cancer patients, there are various reasons why a break in treatment may be necessary. In your opinion, what role do treatment breaks play in defining the line of therapy?
   1. How do you judge this: is the line of therapy considered to have ended after a certain period of time during which the treatment was not continued? If so, how is the length of this period chosen?

**Summary and review**

1. We have now reached the end of the interview. Is there anything else you would like to add?

**Narrative impulse and maintenance questions**

1. The aspect XYZ you just mentioned is very interesting, can you please tell me more about it?
2. You mentioned XYZ. I didn't quite understand that. What/how did you mean that?
3. Can you give me an example of the situation you have described?

**Reflection on the topic of the interview**

1. This aspect is quite interesting, but it takes me away from my actual question. Let's talk about XYZ again.

**Outlook**

That brings us to the end of the interview. Thank you very much for taking the time for this interview with me. You have provided me with a lot of valuable and interesting information. As part of my doctoral thesis, I will transcribe the interview and then analyze it.
